# A novel activation-related gene *P158* is essential for evagination and early development of *Echinococcus multilocularis* protoscoleces

**DOI:** 10.1186/s13071-026-07543-6

**Published:** 2026-07-02

**Authors:** Zhendong Xin, Zhen Wang, Yong Fu, Chenye Jin, Xinyi Zhou, Yihui Wu, Wenjing Zhi, Mengjie Chu, Lijuan Zheng, Anni Zhang, Changjie Qian, Nariaki Nonaka, Ryo Nakao, Zhihong Guo, Gongguan Liu

**Affiliations:** 1 Key Laboratory of Animal Disease Diagnostics and Immunology, Ministry of Agriculture, MOE International Joint Collaborative Research Laboratory for Animal Health & Food Safety, Nanjing Agricultural University, Nanjing, China; 2 Qinghai Provincial Key Laboratory of Pathogen Diagnosis for Animal Diseases and Green Technical Research for Prevention and Control, Academy of Animal Science and Veterinary, Qinghai University, Xining, China; 3 Laboratory of Parasitology, Department of Disease Control, Graduate School of Infectious Diseases, Faculty of Veterinary Medicine, Hokkaido University, Sapporo, Japan; 4 Division of Parasitology, Veterinary Research Unit, International Institute for Zoonosis Control, Hokkaido University, Sapporo, Japan; 5 One Health Research Center, Hokkaido University, Sapporo, Japan

**Keywords:** *Echinococcus multilocularis*, Protoscolex activation, Evaginated protoscolex, RNA interference, Activation-associated gene, *P158* gene

## Abstract

**Background:**

Alveolar echinococcosis is a lethal zoonotic parasitic disease caused by *Echinococcus multilocularis*. The activation and subsequent evagination of protoscoleces (PSCs) in the host digestive tract are critical steps for establishing infection in definitive hosts, yet the underlying molecular mechanisms remain poorly understood.

**Methods:**

In this study, we employed an in vitro PSC activation model combined with transcriptomic analysis to identify activation-associated genes. A novel gene, designated *P158*, was further characterized by transcriptional unit analysis and functional knockdown assays to assess its role in PSC evagination, viability, tegument integrity, energy metabolism, and early metacestode development.

**Results:**

We confirmed that *P158* constitutes an independent transcriptional unit from the adjacent *DDX1* gene of *E. multilocularis* and found that *P158* expression was significantly upregulated following activation. Knockdown of P158 led to a significant reduction in PSC evagination rate and impaired metacestode vesicular development in vitro. Concurrently, decreased cellular viability and damage to the microtriches indicated compromised tegument structural integrity. Further mechanistic analysis showed that *P158* knockdown triggered metabolic dysregulation, characterized by reduced ATP levels, shrinkage of glycogen storage cells, and depletion of intracellular glycogen reserves.

**Conclusions:**

These findings demonstrate that *P158* plays an essential role in promoting PSC evagination and early development by maintaining tegument structural integrity and regulating energy metabolism homeostasis. This study highlights *P158* as a potential target for interventions aimed at blocking parasite development at the definitive host stage.

**Graphical Abstract:**

**Figure:**
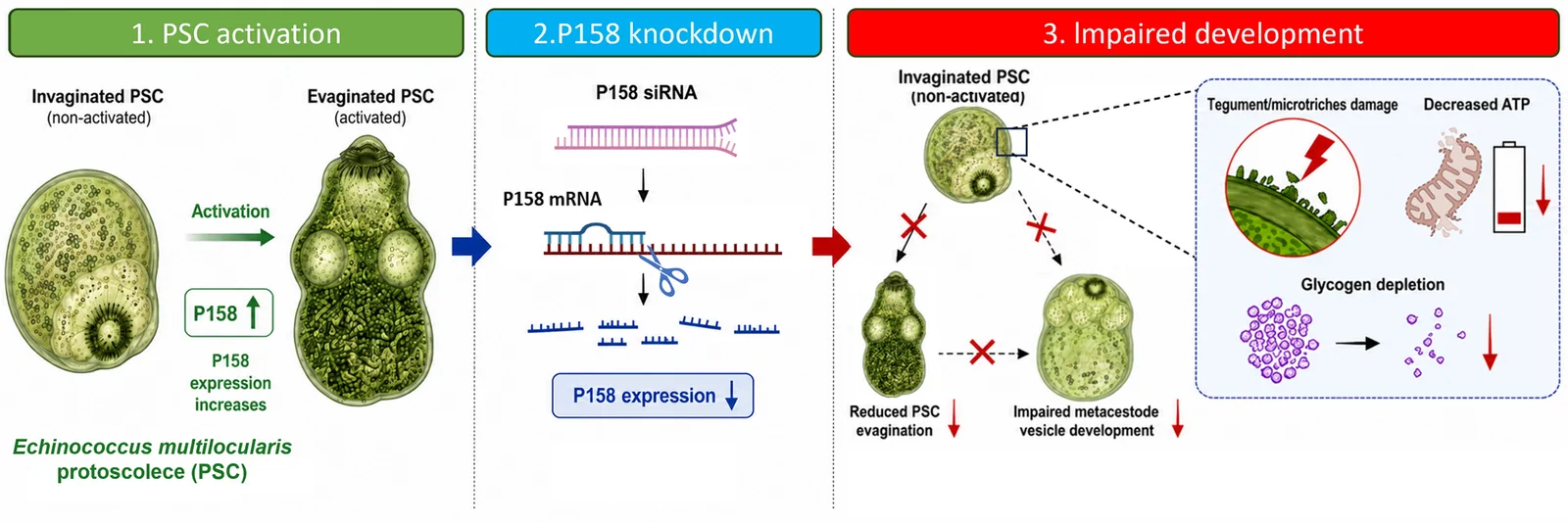

**Supplementary Information:**

The online version contains supplementary material available at https://doi.org/10.1186/s13071-026-07543-6.

## Background

Echinococcosis is a medically important zoonotic parasitic disease resulting from infection by the larval stages of *Echinococcus* species, which poses substantial threats to global public health and livestock productivity [1]. Among the forms of human echinococcosis, primarily cystic and alveolar echinococcosis, alveolar echinococcosis (AE) caused by *E. multilocularis* is considered the most clinically severe, characterized by a tumor-like infiltrative growth pattern and a mortality rate exceeding 90% within 10–15 years in untreated or inadequately treated patients [2, 3]. Current clinical treatments primarily rely on benzimidazoles, which inhibit parasite growth but do not achieve complete eradication. Reports of drug-resistant *E. multilocularis* are rare to date, but long-term administration is associated with significant hepatotoxicity and other adverse effects [4]. Therefore, interrupting the parasite's life cycle in the definitive host represents a critical strategy for transmission control at the source.

Infection by *E. multilocularis* involves a series of critical molecular and cellular events that govern parasite growth, development, and host interactions, ultimately determining pathogenicity within individuals and transmission dynamics between hosts [5–7]. Upon ingestion of infected tissue containing protoscoleces (PSCs) by the definitive host, the PSCs must undergo activation in the digestive tract. This process involves scolex evagination along with morphological and physiological changes, enabling the parasites to attach to the intestinal wall and develop into adult worms, thereby completing the life cycle [1]. In vitro studies demonstrated that chemical stimuli emulating the gastrointestinal environment effectively induce PSC activation, a process crucial for the successful establishment of infection [8, 9].

The molecular regulatory networks underlying PSC activation remain poorly understood, particularly at the levels of gene expression and metabolic regulation, hindering the development of targeted interventions such as novel drugs [10]. With advances in genomics, functional annotation of novel parasite-specific genes identified through comparative omics is commonly performed by assessing sequence homology between *E. multilocularis* and reference organisms such as humans or well-studied model organisms (e.g., mice and the nematode *Caenorhabditis elegans*) [11, 12]. However, this approach has certain limitations that many genes central to parasite infection and development may lack direct orthologs, and that their functions may have undergone specific evolution in the parasite's unique biological context [13].

To elucidate the molecular basis of *E. multilocularis* PSC activation, we integrated the in vitro activation model and the transcriptomic data to systematically compare gene expression profiles before and after activation. This identified a set of activation-related genes, from which we identified a significantly upregulated, functionally uncharacterized gene, tentatively named *P158* for further study. Through RNA interference (RNAi)-mediated KD, we demonstrated for the first time that *P158* is an essential functional gene required for the activation of *E. multilocularis* PSCs.

## Methods

### Parasite preparation and in vitro activation

PSCs of *E. multilocularis* were prepared as described previously [14, 15]. In brief, hydatid cyst tissue was aseptically collected from the peritoneal cavities of Mongolian jirds with secondary AE, established by intraperitoneal infection with *E. multilocularis* PSCs for 5–6 months. The tissue was minced and filtered through sterile 150 μm strainer by washes with sterile phosphate-buffered saline (PBS, pH 7.4) for three times. For PSCs purification, the suspension was allowed to settle by gravity in centrifuge tubes. The supernatant, containing cyst wall debris and residual tissue, was then aspirated and discarded.

For comparative in vitro assays, PSC activation was induced using an established pepsin-based protocol [5, 14]. Briefly, PSCs were resuspended in pre-warmed 0.05% pepsin (Sigma, USA) solution (dissolved in Hanks' balanced salt solution, pH 2.0) and incubated at 37 °C for 30 min. Control (non-activated) PSCs were incubated under identical conditions in an equal volume of PBS (pH 7.4). After incubation, both groups were washed three times with PBS to terminate the reaction. PSC viability post-activation was assessed by trypan blue staining [16]. At least 200 PSCs were randomly counted per group, and only preparations with initial viability > 95% were used for subsequent experiments. Both activated and non-activated PSCs were maintained in vitro at 37 °C with 5% CO₂ in complete DMEM (Bio-Channel, China) medium supplemented with 20% fetal bovine serum (FBS, EXCEL, China), 50 U/mL penicillin, and 50 μg/mL streptomycin. All pipette tips used during procedures were pre-coated with FBS to prevent PSC adhesion and loss.

### Assessment of activation phenotype and evagination rate

PSC activation status was evaluated primarily based on evagination rate (ER) [17]. Under an inverted fluorescence microscope (MF52-N; MSHOT, Guangzhou, China) (40 × objective), at least three random fields of view (each containing no fewer than 100 PSCs) were selected, and the numbers of invaginated and evaginated PSCs were manually counted. The ER was calculated using the following formula:

$${\text{ER }}\left( \% \right)\, = \,\left[ {{\text{number of evaginated PSCs}}/\left( {{\text{number of invaginated PSCs}}\, + \,{\text{number of evaginated PSCs}}} \right)} \right]\, \times \,{1}00\% .$$

Each experimental group included three independent biological replicates, and the results are presented as mean ± standard deviation (SD).

### Screening of activation-related differentially expressed genes

To systematically identify genes upregulated during PSC activation, we re-analyzed a publicly available transcriptome dataset reported by Herz et al. (Dataset S5) [18]. This dataset includes genes with expression abundance in PSCs significantly higher than those in metacestode vesicles (PS/MC ratio > 10), with expression levels represented as median transcripts per million (TPM). TPM values for non-activated PSCs (PS_nonact) and activated PSCs (PS_act) were extracted from the dataset. Fold change (FC) in expression for each gene was calculated as FC = TPM_PS_act/TPM_PS_nonact. Genes with FC > 2.0 were defined as "activation-specifically upregulated genes" [19], yielding 103 candidates (see Table S1). To visualize expression patterns, scatter plots of TPM values for PS_act (PA) versus PS_nonact (PNA) were generated using OriginPro 2023 software (OriginLab, USA). To accommodate the wide dynamic range of expression levels, gene expression values were visualized using a scatter plot with log_10_ scaled axes.

### Gene cloning and transcriptional unit analysis

Total RNA was extracted from PSCs using TRIzol reagent (Takara Bio, Beijing, China) according to the manufacturer's instructions. First-strand cDNA synthesis was performed using the Hifair^®^ II 1st Strand cDNA Synthesis SuperMix for qPCR (gDNA digester plus) kit (Yeasen, Shanghai, China) following the provided protocol. Specific primers were designed based on gene sequences from the WormBase ParaSite database (genome assembly PRJEB122) (*P158*: EmuJ_000545500; *DDX1*: EmuJ_000545600) to amplify full-length coding sequences. Primers for *P158* were *P158*F (5′-ATGGGGGTGTGGTTCTTAGCG-3′) and *P158*R (5′-TGGGGTGGAATAAGGGGGAGG-3′); primers for *DDX1* were *DDX1*F (5′-ATGGGAACAGCTTTCGAAACTGAA-3′) and *DDX1*R (5′-ACACTTAGCTGCACCGCCATATCG-3′). PCR amplification was carried out using 2 × Phanta Max Master Mix (Vazyme, Nanjing, China). The reaction program consisted of initial denaturation at 95 °C for 5 min, followed by 35 cycles of 95 °C for 30 s, 58 °C for 30 s, and 72 °C for 1 min, with a final extension at 72 °C for 5 min.

To further validate genomic structure and obtain flanking sequences, additional PCR primers were designed for touchdown PCR amplification. The touchdown PCR conditions were as follows: initial denaturation at 95 °C for 5 min, followed by 10 touchdown cycles consisting of denaturation at 95 °C for 30 s, annealing (starting at 66 °C and decreasing by 2 °C every two cycles until 56 °C), and extension at 72 °C for 1 min; subsequently, 30 standard cycles with annealing at 56 °C, and a final extension at 72 °C for 5 min. The specific primers used for amplification were as follows: *P158*F1 (5′-TTCTACTTATCGCTAGCGTTTATGAGG-3′), *P158*F2 (5′-TGAGCATTGGATTGTTGGTATATTTCTG-3′), *P158*F3 (5′-TTTACCCCCACCCAGCATTC-3′), *P158*F4 (5′-TCCTCCCCCTTATTCCACCCCA-3′), *DDX1*R1 (5′-GACTTCTTTCCCGTACCGCCAAAA-3′), *DDX1*R2 (5′-AATTTCCTCCGTTAGTTCCCTGG-3′), *DDX1*R3 (5′-AACACCGTCAGTCTGGACATG-3′), and *DDX1*R4 (5′-TGCCAACACGACCAATACGATG-3′).

To confirm the genomic positions of *P158* and *DDX1*, BLAST searches were conducted using the WormBase ParaSite database tool (E-value cutoff < 1 × 10⁻^5^) against the *E. multilocularis* genome. The regions encompassing these genes were identified as pathogen_EmW_scaffold_07:4,774,559–4,779,675 (*P158*) and pathogen_EmW_scaffold_07:4,781,627–4,795,656 (*DDX1*). The DNA sequence of the extended region spanning both genes and the intergenic spacer was retrieved and submitted to the NCBI ORFfinder tool (https://www.ncbi.nlm.nih.gov/ORFfinder/). ORF prediction was performed with the following parameters: ATG as the only start codon and minimum ORF length of 75 nucleotides. This analysis aimed to determine whether *P158* and *DDX1* function as independent transcriptional units. Genomic structures, ORF predictions, and gene alignments were visualized using the IBS 2.0 online tool (https://ibs.renlab.org/) and SnapGene software (version 6.0.2, GSL Biotech). This ORF analysis was performed on the *P158*–*DDX1* intergenic spacer to evaluate whether it could support a continuous coding sequence linking the two loci, rather than to re-annotate the known *P158* coding sequence.

### RNA interference and functional phenotype assessment

Each biological replicate consisted of protoscoleces isolated from an independent experimentally infected host at 6 months post-infection. Specific small interfering RNA targeting *P158* (si*P158*: 5′-GUACAAGACGUUCCGUCAAAT-3′) and negative control siRNA (NC-siRNA: 5′-UUCUCCGAACGUGUCACGU-3′) were synthesized by Tsingke Biotechnology Co., Ltd. (Beijing, China). The specificity of both siRNAs was evaluated by BLASTn-short searches against the *E. multilocularis* PRJEB122 genome assembly EMULTI002 (GCA_000469725.3) available in WormBase ParaSite. Before BLASTn analysis, RNA sequences were converted to DNA sequences by replacing U with T. The following parameters were used: word size = 7, expect threshold = 1000, dust filtering disabled, and both strands searched. Potential off-target sites were defined as ungapped full-length alignments with ≤ 3 mismatches. No potential off-target sites were detected for the NC-siRNA. Prior to RNAi transfection, the purified PSCs were subjected to the in vitro activation treatment as described above. RNAi transfection was performed by electroporation [20]. Briefly, the electroporation procedure was as follows: approximately 5,000 PSCs were resuspended in 200 μL electroporation buffer (150 mM sucrose, 27 mM Na₂HPO₄, pH 7.5), and siRNA was added to a final concentration of 50 nM. Transfection was performed in 2 mm cuvettes using a Bio-Rad Gene Pulser electroporator with square-wave pulses at 125 V for 20 ms. After transfection, PSCs were incubated at 37 °C for 30 min to recover, followed by addition of 1 mL complete medium and transfer to 6-well plates for further culture. Culture supernatants and PSCs were then collected at 72, 120, and 168 h post-transfection.

Total RNA was extracted for RT-qPCR to quantify relative mRNA expression levels of *P158* and *DDX1*, thereby assessing KD efficiency; ER and cyst formation rates (Cyst rate, CR; defined by whether the scolex develops into vesicle stage) were analyzed under a light microscope as described previously [21]; cell viability was evaluated using fluorescein diacetate (FDA)/propidium iodide (PI) double staining and observed under the MF52-N inverted fluorescence microscope [17]; Phosphoglucose isomerase (PGI) activity in culture supernatants was measured as a marker of cell damage using a colorimetric assay kit (Beyotime) [22]; PSCs were collected, total protein concentration determined by BCA assay for normalization, and intracellular ATP levels quantified using an enhanced ATP assay kit (Beyotime).

### Reverse transcription quantitative PCR (RT-qPCR)

Total RNA was extracted from PSCs following different treatments. RNA concentration was measured using an ND-1000 spectrophotometer (Thermo Fisher Scientific, US), and approximately 1 μg of total RNA from each sample was used for reverse transcription into cDNA. RT-qPCR was performed on a Roche LightCycler 96 system using Hieff UNICON^®^ Universal qPCR SYBR Green Master Mix (Yeasen). The 20 μL reaction volume consisted of 10 μL master mix, 2 μL cDNA template, 1 μL (0.4 μM) each forward and reverse primer, and 6 μL nuclease-free water. Primers were as follows: for *P158*, *P158*F (5′-TGTCTTCATCGTCGTGGCAA-3′) and *P158*R (5′-GAATGCTGGGTGGGGGTAAA-3′); for *DDX1*, *DDX1*F (5′-AACGGCGATGTCAACTTCCT-3′) and *DDX1*R (5′-TGCCAACACGACCAATACGA-3′); for the reference gene (*β-actin*), *β-actin*F (5′-CCAAGGCGAACCGTGAAAAG-3′) and *β-actin*R (5′-GTTCGACCAGAGGCGTAGAG-3′). The cycling program was as follows: initial denaturation at 95 °C for 5 min, followed by 40 cycles of 95 °C for 10 s, 60 °C for 30 s, and 72 °C for 20 s. Each sample was run in three technical replicates. The amplification efficiencies of the target and reference genes were evaluated using standard curves and found to be approximately equal (E ~ 100%), justifying the use of the 2^−ΔΔCq^method for the calculation of relative expression levels of target genes [23].

### Scanning electron microscopy (SEM) and transmission electron microscopy (TEM) sample preparation and observation

To examine the effects of *P158* KD on the ultrastructure of the PSC surface, approximately 500 PSCs from each experimental group were collected and washed with PBS. Samples were fixed overnight at 4 °C in 2.5% glutaraldehyde [24]. Fixed PSCs were then rinsed three times with ultrapure water (10 min each), followed by sequential dehydration in an ethanol gradient (30%, 50%, 70%, 80%, and 90% ethanol for 15 min each) and three changes of 100% ethanol (30 min each). Ethanol was subsequently replaced with tert-Butanol in four changes (30 min each). After replacement, samples were placed on glass coverslips. Following freeze-drying, PSCs were mounted and gold-sputter coated prior to observation under a Hitachi Regulus 8100 scanning electron microscope (Hitachi, Tokyo, Japan).

After gradient dehydration, samples were infiltrated with epoxy resin. Infiltrated samples were placed in capsules or embedding molds, embedded with embedding medium and epoxy resin, and sectioned. Sections were double-stained with uranyl acetate and lead citrate, dried at room temperature, and observed using a Hitachi HT7800 transmission electron microscope (Hitachi, Tokyo, Japan).

### RNA sequencing and data analysis

Following siRNA transfection, PSCs from the *P158* KD and NC groups were cultured in vitro for 72 h as described above. At this time point, approximately 5,000 PSCs were randomly collected from each experimental group (three biological replicates per group). The collected PSCs were centrifuged, washed with pre-chilled PBS containing penicillin and streptomycin, and centrifuged again to remove the supernatant. The resulting pellets were snap-frozen in liquid nitrogen and submitted to Tsingke Biotechnology Co., Ltd. (Beijing, China) for total RNA extraction, library preparation, and high-throughput sequencing on the Illumina NovaSeq 6000 platform (paired-end 150 bp reads).

Raw sequencing data were processed for quality control, mapping to the *E. multilocularis* reference genome, and transcript assembly using standard pipelines. Crucially, differential expression analysis was performed using the DESeq2 package (v1.26.0) [25], and GO/KEGG enrichment analyses were conducted using clusterProfiler (v3.14.0) [26]. All analyses and visualizations were carried out via the Tsingke Cloud Platform (Tsingke Biotechnology Co., Ltd., Beijing, China).

### Statistical analysis

Statistical analyses were performed using GraphPad Prism software (version 6.0). Group comparisons were conducted using two-sided Student’s t-test. For transcriptomic analyses, including differential expression and GO/KEGG enrichment analyses, *p*-values were adjusted using the Benjamini–Hochberg procedure to control the false discovery rate (FDR), because multiple genes or pathways were tested simultaneously. Unless otherwise specified, statistical significance was defined as *p* < 0.05.

## Results

### In vitro activation and morphological assessment of PSCs

**Fig. 1:**
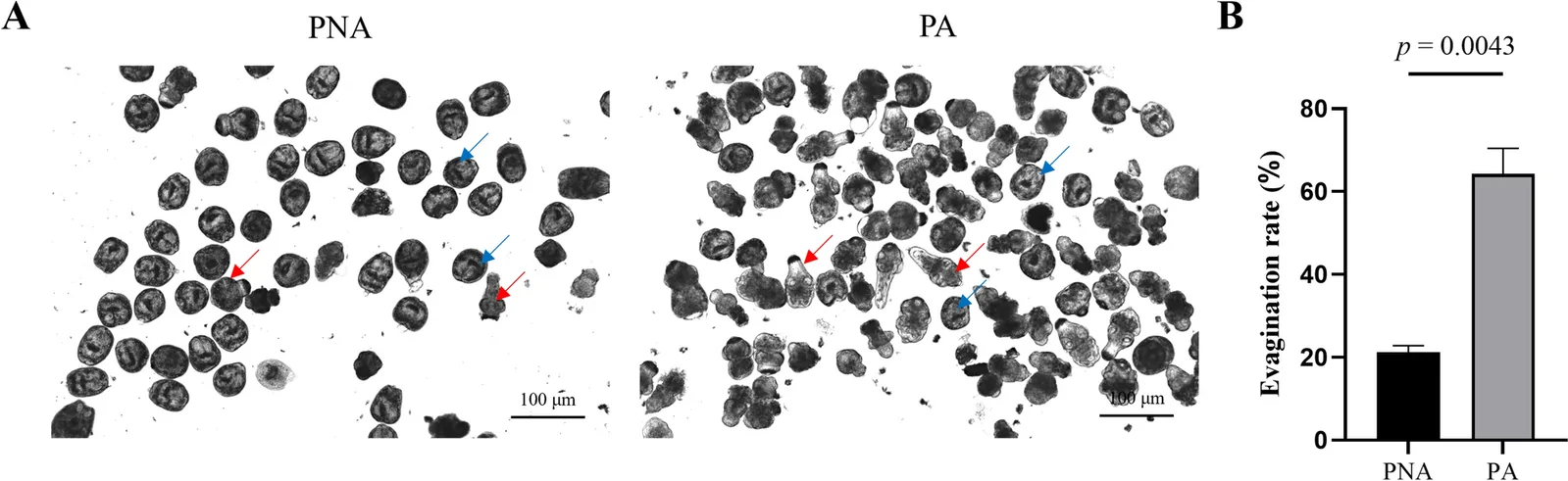
In vitro activation and evagination of *E. multilocularis* protoscoleces. **A** Morphological comparison of protoscoleces (PSCs) after 72 h cultivation. Non-activated PSCs (PNA) were treated with PBS, while activated PSCs (PA) were treated with pepsin solution. Red arrows indicate evaginated PSCs; blue arrows indicate non-evaginated PSCs. **B** Quantification of evagination rates. Data are presented as mean ± SD (*n* = 3)

A pepsin-based stimulation was applied as a defined in vitro activation condition to induce PSC evagination [27, 28]. Upon in vitro culture following pepsin exposure, PSCs displayed pronounced morphological alterations, characterized by prominent scolex evagination and body segment elongation (Fig. 1A). In contrast, non-activated PSCs primarily displayed an invaginated scolex morphology, although a subset of PSCs underwent spontaneous evagination during the 72 h in vitro culture even without pepsin stimulation (Fig. 1A). Quantitatively, after 72 h of in vitro culture, the pepsin-activated group (PA) showed a significantly higher evagination rate than the PBS-treated non-activated control group (PNA), with a difference exceeding threefold (PNA: 20.9% ± 1.65% vs. PA: 64.3% ± 6.11%; *n* = 3; Fig. 1B). These observations confirm that gastrointestinal chemical stimuli effectively promote the transition of PSCs from a dormant, invaginated state to an active, evaginated morphology.

### Screening and analysis of activation-related genes

**Fig. 2:**
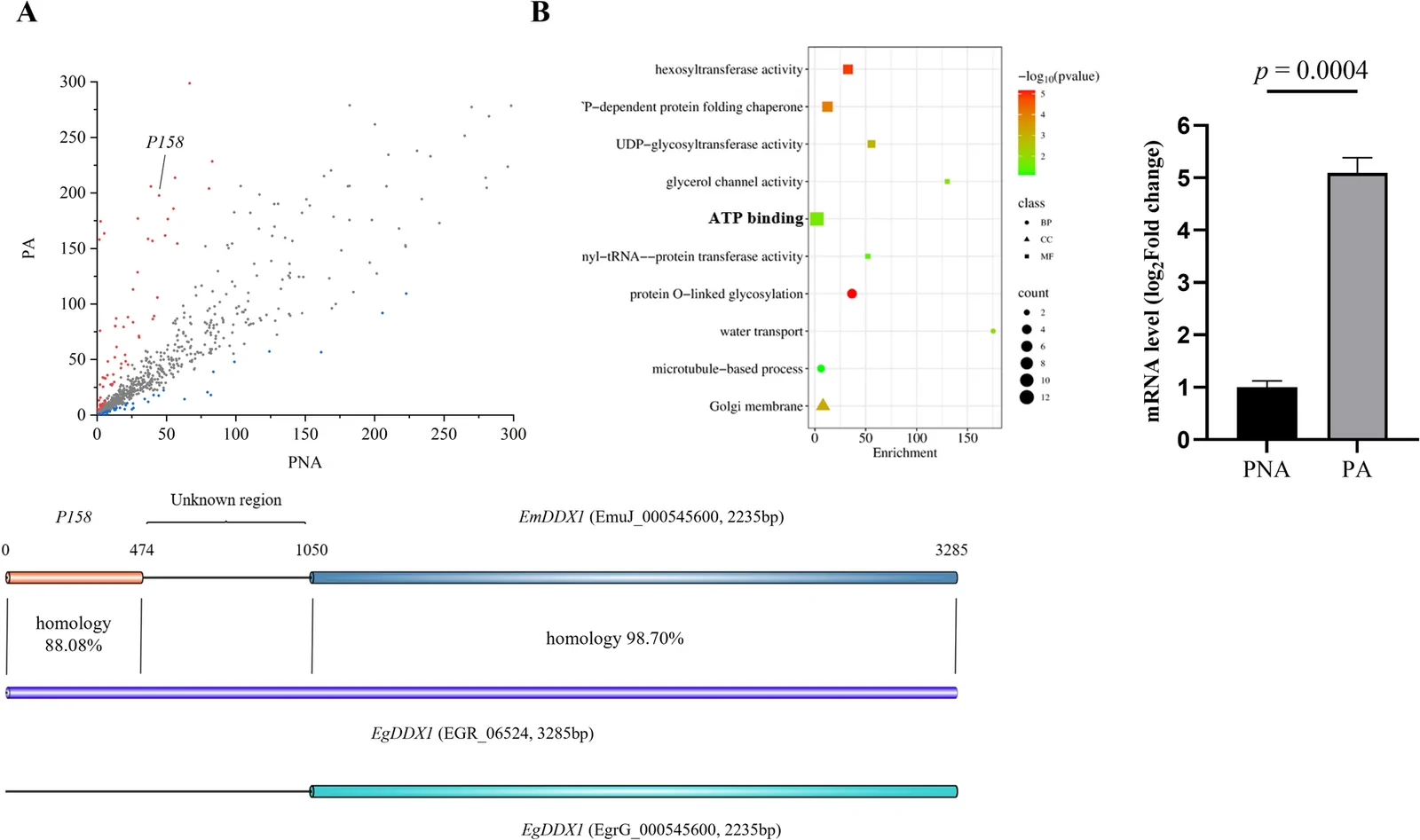
Transcriptomic analysis and identification of the activation-associated gene *P158*. **A** Scatter plot of transcript per million (TPM) values for PSC-specific genes in activated (PA, y-axis) versus non-activated (PNA, x-axis) PSCs, based on re-analysis of a published transcriptomic dataset. Genes with a fold change > 2 are highlighted in red (*n* = 103), of which the candidate gene *P158* (EmuJ_000545500.1) is indicated. **B** Validation of *P158* upregulation by quantitative RT-PCR in PSCs of PA and PNA groups. Data are presented as mean ± SD (*n* = 3; normalized to *β-actin*). **C** Schematic representation of the genomic loci of *P158* (EmuJ_000545500.1) and *EmDDX1* (EmuJ_000545600) in *E. multilocularis* (top, assembly PRJEB121), aligned with two distinct genomic annotations of *EgDDX1* in *E. granulosus*: the fused transcript version EGR_06524 (middle, assembly PRJNA182977) and the independent transcript version EgrG_000545600 (bottom, assembly PRJEB121)

To identify differentially expressed activation-related genes, we first conducted an in-depth re-analysis of a published transcriptome dataset comparing activated and non-activated PSCs [18, 29]. This study identified 872 genes with significantly higher expression in PSC than in metacestode vesicles (expression > tenfold higher). From this gene set, we further compared expression levels between activated and non-activated states. We herein identified 103 significantly upregulated genes (fold change > 2) and 57 significantly downregulated genes following activation (Fig. 2A). Notably, a substantial proportion of these upregulated genes were annotated as "expressed protein" or "expressed conserved protein" (Table S1).

To identify genes potentially involved in the activation of *E. multilocularis* (Em) PSCs, we performed the NCBI BLAST homology analysis of the 103 hits and revealed that one "expressed protein," designated *P158*, showed high similarity to the ATP-dependent RNA helicase *DDX1* from *E. granulosus*, with 88.08% nucleotide sequence identity (EGR_06524) and 84.11% amino acid sequence identity (W6UCZ3) (Fig. S1). To verify this transcriptomic data, we then performed RT-qPCR validation and confirmed a significantly higher expression of *P158* in activated PSC compared to the non-activated group (Fig. 2B).

At the genomic level, P158 (WormBase ID: EmuJ_000545500.1; pathogen_EmW_scaffold_07: 4,774,559–4,779,675) is located immediately upstream of *EmDDX1* (WormBase ID: EmuJ_000545600; pathogen_EmW_scaffold_07: 4,781,627–4,795,656) on the same scaffold and strand, separated by only 576 nucleotides (Fig. 2C). In contrast, in the *E. granulosus* reference genome (assembly PRJNA182977), the corresponding homologous sequences are annotated as a single fused transcript (*EgDDX1*, EGR_06524). We therefore hypothesized that this discrepancy may represent an annotation artifact, wherein a single transcript was potentially split into separate entries [30]. Under this hypothesis, the *P158* gene sequence may correspond to the 5' extension of the *EmDDX1* transcript, aligning with the structure of *E. granulosus EgDDX1* (EGR_06524) (Fig. 2C middle). Alternatively, *P158* and *EmDDX1* could function as mutually independent transcriptional units, consistent with the annotation of *E. granulosus EgDDX1* (EgrG_000545600) (Fig. 2C bottom).

### *P158* and *DDX1* constitute independent transcriptional units

**Fig. 3:**
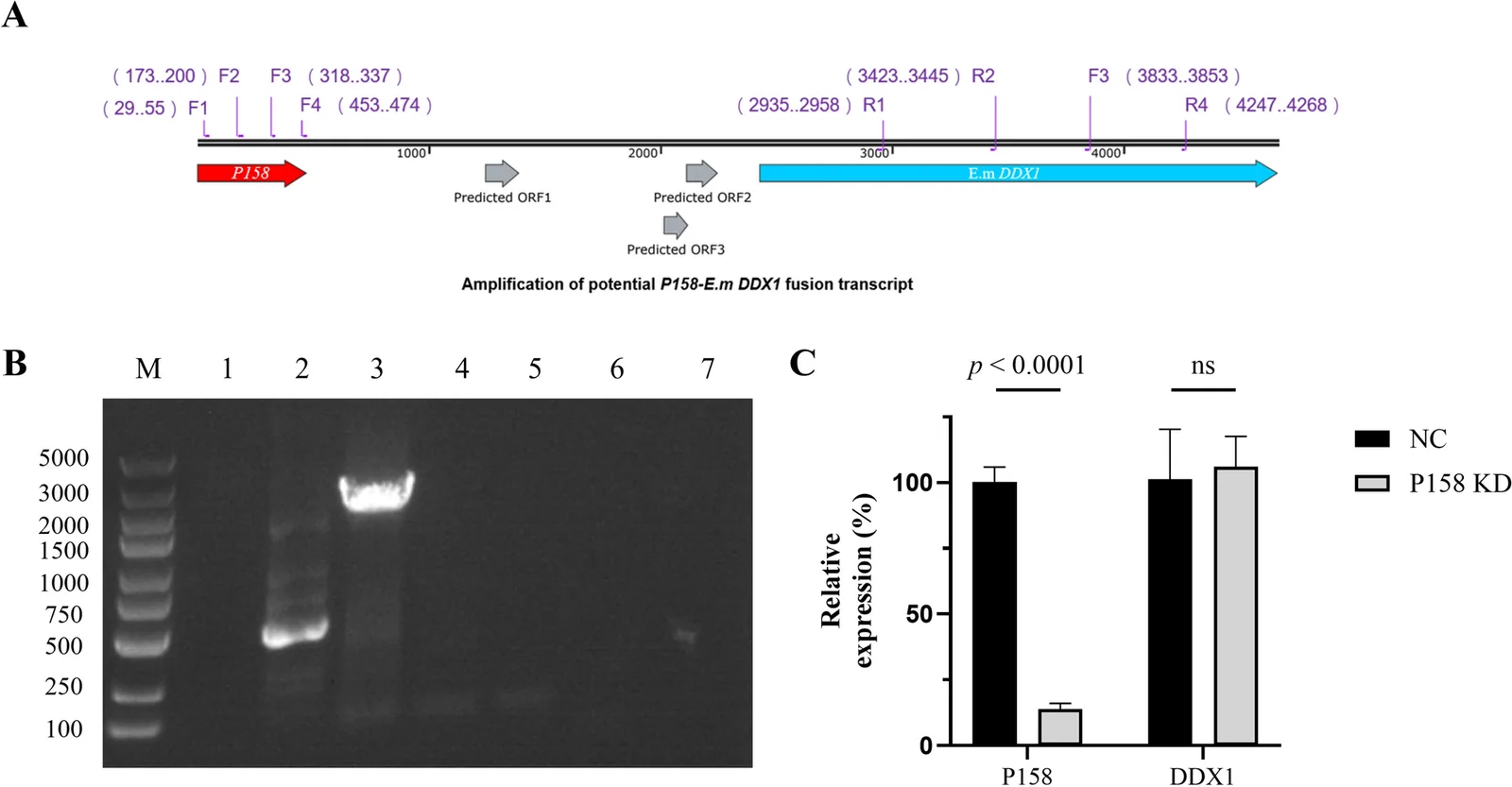
Validation of *P158* as an independent transcriptional unit in *E. multilocularis*. **A** Open reading frame (ORF) prediction of the 1,954-nucleotide intergenic region between EmuJ_000545500.1 (*P158*, red arrow) and EmuJ_000545600.1 (*EmDDX1*, blue arrow). Forward (F1–F4) and reverse (R1–R4) primers designed for potential fusion transcript amplification are indicated. **B** Agarose gel electrophoresis of RT-PCR products. Lanes: M, DNA ladder with molecular sizes indicated on the left of the gel image (5000, 3000, 2000, 1500, 1000, 750, 500, 250, and 100 bp); (1) NC (no template); (2) *P158*-specific amplicon (477 bp); (3) *DDX1*-specific amplicon (2235 bp); lanes 4–7, amplification attempts using primer pairs spanning the intergenic region (expected products > 2,712 bp; none detected). **C** Quantitative RT-PCR analysis of *P158* and *DDX1* expression at 72 h post-transfection for siRNA-mediated KD of *P158*. Data are presented as mean ± SD of relative expression normalized to *β-actin* (*n* = 3). *ns* not significant (*P* > 0.05). *NC* negative control transfected with irrelevant dsRNA

To clarify the relationship between *P158* and *DDX1*, we first analyzed the genomic spacer region between them. ORF prediction using the NCBI ORFfinder tool identified 7 open reading frames (> 75 nucleotides) on the forward strand of the 1.95 kb spacer region. The longest ORF (ORF1) was 144 nucleotides (47 amino acids), with the remaining ORFs not exceeding 135 nucleotides (Figs. 3A, S2). BLAST analysis did not detect significant similarity of these short ORFs to annotated proteins (*E* > 10^–2^). Importantly, these ORFs did not provide evidence for a continuous protein-coding sequence linking *P158* and *DDX1*, supporting the interpretation that the two genes do not form a fused coding unit.

To confirm transcriptional independence at the mRNA level, specific primers were designed. RT-PCR results demonstrated successful amplification of the expected bands for *P158* (477 bp) and *DDX1* (2235 bp) from PSC cDNA (Fig. 3B, lanes 2 and 3). To rule out the existence of a fusion transcript, multiple primer pairs spanning the intergenic region were tested using touchdown PCR; no products were detected across all combinations (Fig. 3B, lanes 4–7), with consistent results in three biological replicates. These findings suggest that *P158* and *DDX1* are transcribed independently, with no evidence of a fusion transcript.

To further confirm the independence of *P158* at the level of expression regulation, we introduced *P158*-specific siRNA into PSCs via electroporation, achieving efficient KD of *P158* (inhibition rate > 80%). Notably, *P158* KD had no significant effect on *DDX1* expression (Fig. 3C). These results, spanning genomic structure, transcript form, and expression regulation, collectively demonstrate that *P158* is an independent transcriptional unit in the *E. multilocularis* genome, rather than a 5' extension of *DDX1*.

### *P158* KD inhibits in vitro activation of PSCs

**Fig. 4:**
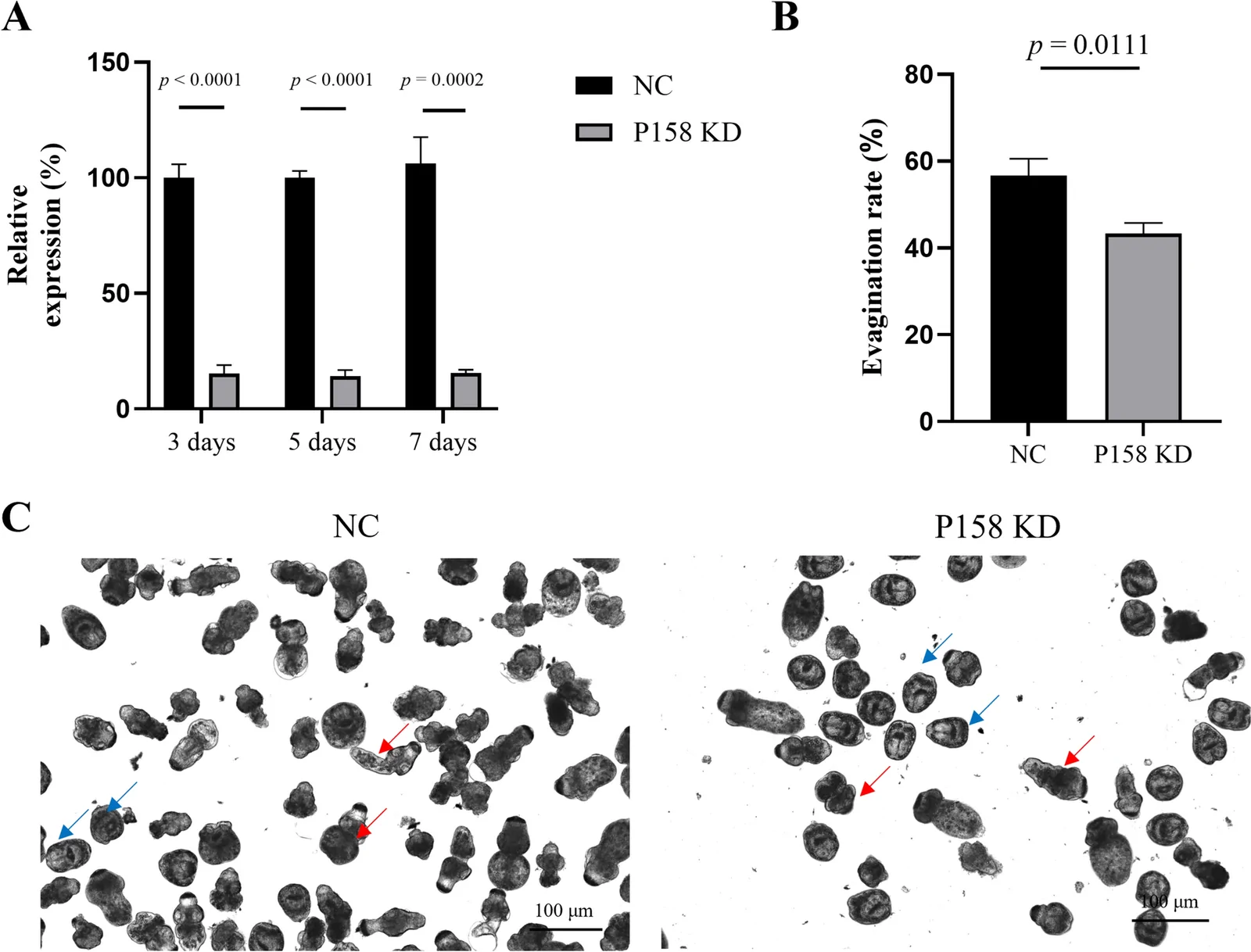
Knockdown of *P158* impairs PSC activation. **A** Time-course analysis of *P158* expression by qPCR following siRNA transfection, showing sustained knockdown (KD) from 72, 120, and 168 h post-transfection. Data are presented as mean ± SD (*n* = 3). **B** Evagination rates of *P158* KD and negative control (NC) PSCs after 72 h in culture. Data are presented as mean ± SD (*n* = 3, > 200 PSCs per group). **C** Representative light microscopy images. NC PSCs exhibit normal evagination, whereas P158 KD PSCs predominantly remain in a non-evaginated state. Red and blue arrows indicate evaginated and non-evaginated PSCs, respectively

Following the successful suppression of *P158* expression via siRNA, we systematically evaluated its impact on in vitro PSC activation. RT-qPCR data confirmed that electroporation-mediated transfection of specific siRNA achieved efficient and sustained KD of *P158*. At 72, 120, and 168 h post-transfection, *P158* expression levels were significantly suppressed to 12–19% relative to the negative control group (NC) (Fig. 4A). Consistent with the gene expression changes, *P158* KD significantly impaired PSC activation. At 72 h post-transfection, the scolex evagination rate in the KD group was markedly lower than that in the NC group (Fig. 4B). Morphological observations further confirmed that PSCs of the NC group exhibited typical evaginated activated morphology, whereas the majority in the *P158* KD group remained in invaginated state (Fig. 4C). These results indicate that *P158* is a key regulatory gene governing the transition of PSCs from a dormant to an activated state.

### *P158* KD disrupts PSC cellular structure and developmental capacity

**Fig. 5:**
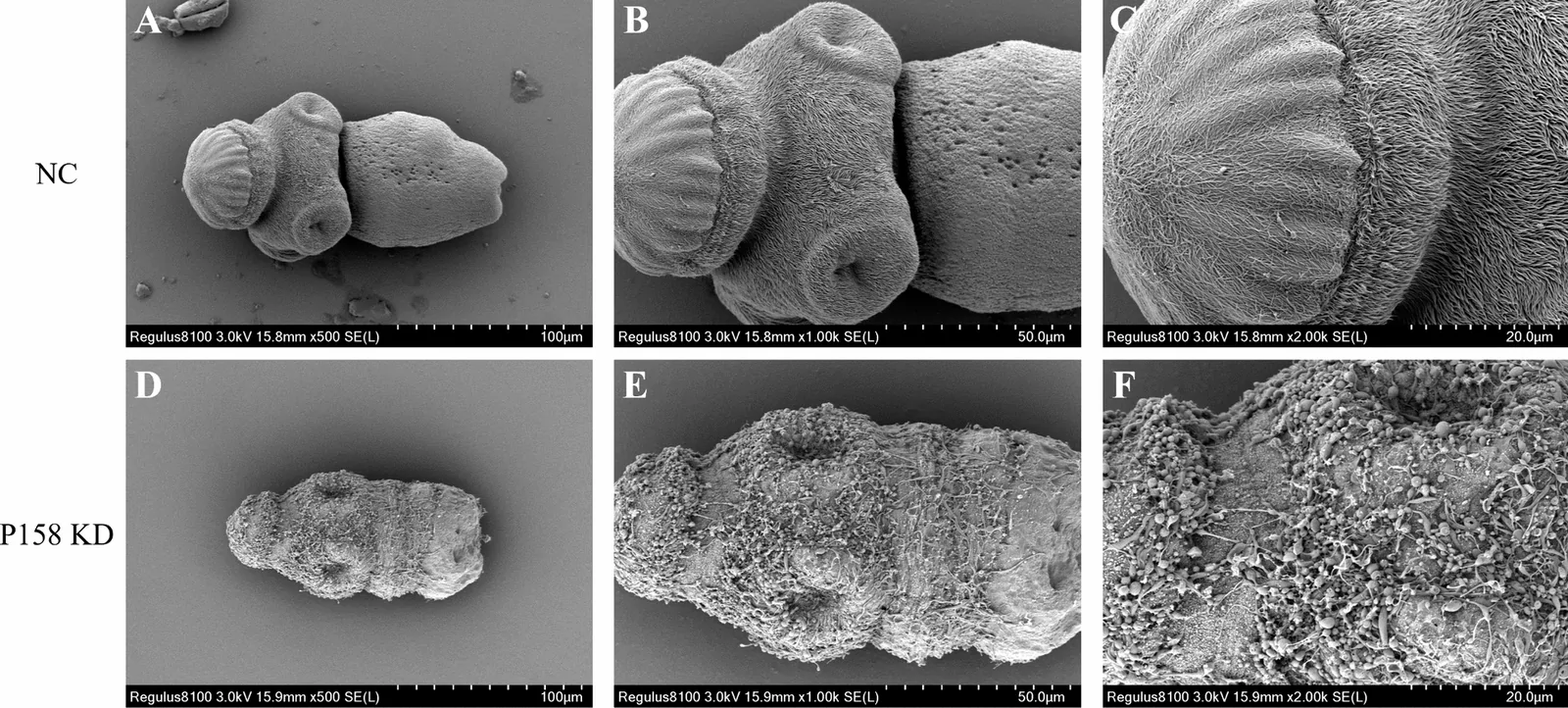
Ultrastructural damage in PSCs following *P158* KD. Scanning electron microscopy (SEM) images of PSCs after 168 h of cultivation. **A**–**C** NC group: overall morphology (**A**), intact tegument with abundant microtriches (**B**), higher magnification of the rostellar region (**C**). **D**–**F** P158 KD group: overall morphology showing body contraction and surface damage (**D**), severe loss of microtriches and accumulation of surface debris (**E**), higher magnification revealing disrupted tegument integrity (**F**)

To evaluate the long-term effects of *P158* KD on parasite structure and cellular health, analyses were performed at 168 h post-transfection. Scanning electron microscopy (SEM) results showed that the surface structure of NC group PSCs was intact, with the rostellum and suckers densely covered by well-preserved microtriches (Fig. 5A–C) [31]. In contrast, the *P158* KD group exhibited marked morphological abnormalities and structural damage, including body contraction, compromised tegument integrity, extensive microtriches loss, and accumulation of abnormal surface secretions (Fig. 5D–F). These findings indicate that *P158* plays a crucial role in maintaining tegument structure and surface integrity of PSCs.

**Fig. 6:**
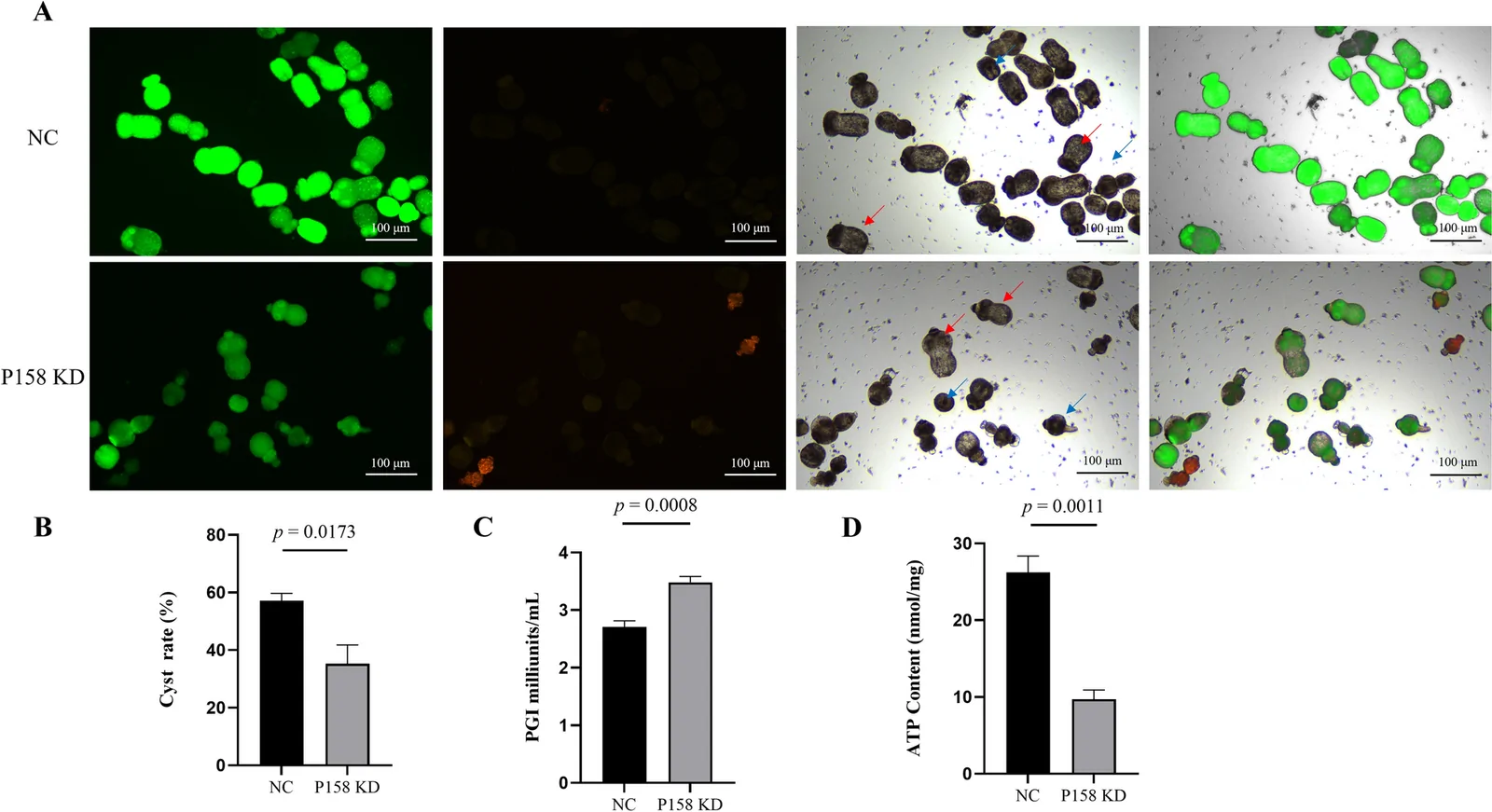
*P158* KD compromises cellular viability, development, and energy metabolism. **A** Viability assessment by FDA/PI staining at 168 h post-transfection. Images of FDA fluorescence (green, live cells), PI fluorescence (red, dead cells), brightfield, and merged overlay are arranged from left to right. The NC group exhibits robust viability with predominant green fluorescence, whereas the P158 KD group shows reduced viability with increased PI-positive cells. **B** Cyst formation rate (percentage of PSCs developing into metacestode vesicles) at 168 h post-transfection. Data are presented as mean ± SD (*n* = 3, ≥ 100 PSCs per group). **C** Phosphoglucose isomerase (PGI) activity in culture supernatants at 168 h post-transfection, indicating increased cellular leakage. Data are presented as mean ± SD (*n* = 3). **D** Intracellular ATP levels at 168 h post-transfection. Data are presented as mean ± SD from three independent biological replicates, with PSCs isolated from independent experimentally infected jirds. *P* = 0.0011, uncorrected two-sided Student’s *t*-test

To further corroborate the association between structural damage and developmental defects, we performed the FDA/PI double staining and revealed a marked decrease in cell viability in PSCs upon *P158* KD (Fig. 6A). Additionally, vesicle formation rate, a key indicator of further developmental capacity under culture conditions, assessed by the emergence of the characteristic posterior bladder (gourd-like swelling) was significantly reduced in the *P158* KD group compared to that of the NC group (Fig. 6B), indicating that *P158* silencing impedes the in vitro developmental progression of PSCs into metacestode vesicles. Concurrently, release of the cell damage marker phosphoglucose isomerase (PGI) into culture supernatants was significantly elevated in the KD group (Fig. 6C), directly evidencing increased cell membrane permeability or exacerbated injury and disruption of cellular homeostasis.

### PSCs with *P158* KD exhibit energy metabolism disruption

Given the marked decrease in cell viability and the failure of highly energy-dependent developmental processes (i.e., scolex evagination and metacestode vesicle formation), we hypothesized that *P158* silencing might compromise fundamental energy homeostasis within the parasites. To investigate this, we examined the impact of *P158* KD on intracellular ATP levels, and our results showed an approximately 60% reduction in intracellular ATP levels in the KD group compared to that of the NC group (NC: 26.2 ± 2.2 nmol/mg vs. KD: 9.71 ± 1.21 nmol/mg; Fig. 6D). This severe ATP depletion indicates that *P158* KD profoundly impairs mitochondrial function or glycolytic pathways critical for energy production. Such energy metabolism crisis may not only directly constrain energy-intensive processes like activation and motility but also exacerbate cellular homeostasis collapse, forming a vicious cycle.

**Fig. 7:**
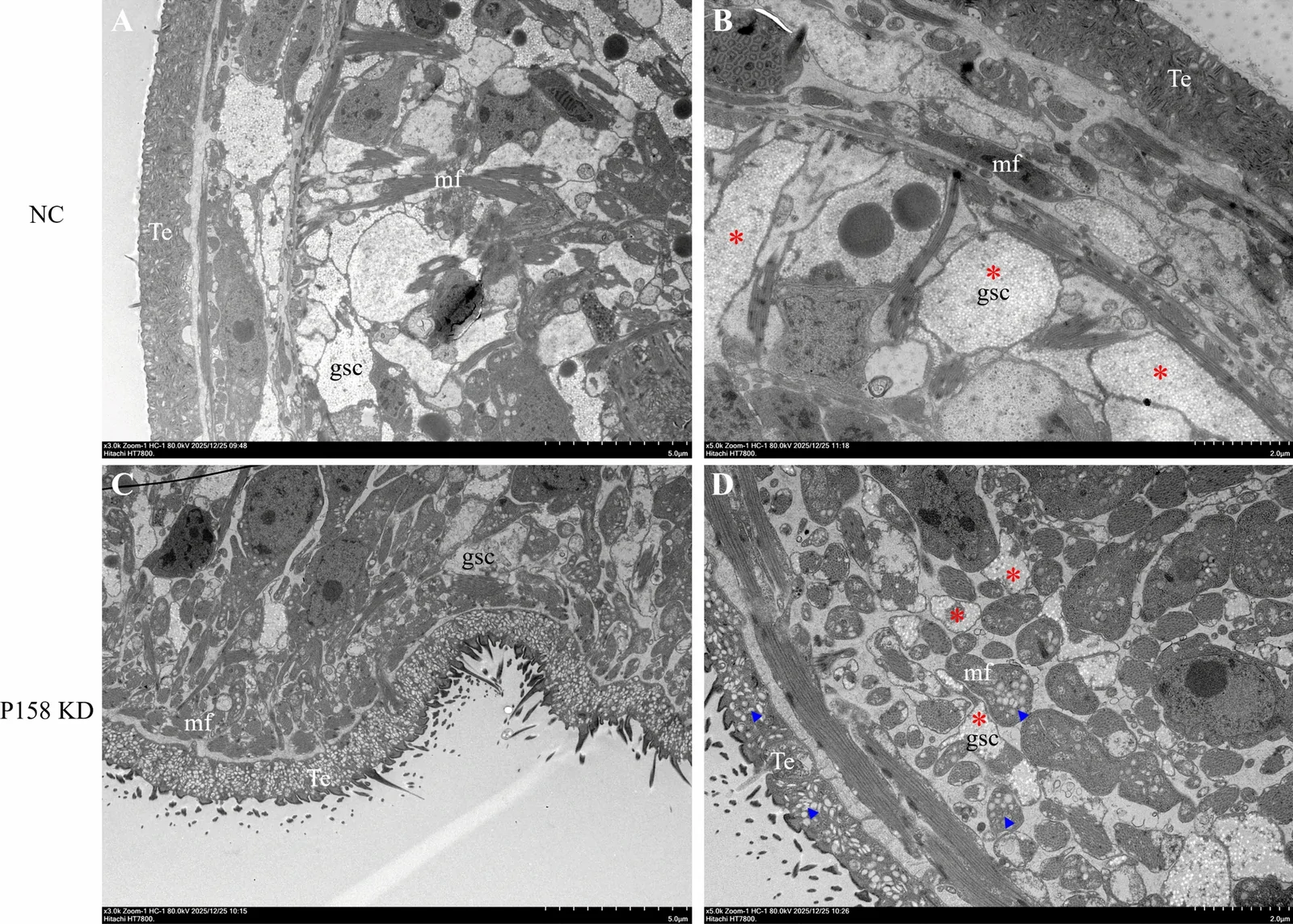
Transmission electron microscopy (TEM) revealing the association of glycogen depletion in PSCs with ultrastructure damage upon *P158* KD. Transmission electron microscopy (TEM) images of PSCs at 168 h post-transfection. **A**, **B** Representative TEM images of NC PSCs showing intact tegument (Te), well-organized muscle fibers (mf), and glycogen storage cells (gsc, red asterisks) with abundant glycogen reserves. **C**, **D** TEM images of P158 KD PSCs revealing extensive vacuolization (indicated by blue arrow heads) in both the tegument (Te) and muscle fibers (mf), along with shrinkage and glycogen depletion in storage cells (gsc, red asterisks)

To uncover the ultrastructural basis of this energy metabolism disruption, transmission electron microscopy (TEM) was used to assess morphological changes in PSCs upon *P158* KD. In the NC group, the tegument (Te) appeared intact with a dense, continuous structure, muscle fibers (mf) were well organized, and glycogen storage cells (gsc) exhibited abundant intracellular glycogen reserves (Fig. 7A, B) [32]. In contrast, the *P158* KD group displayed marked pathological alterations, including extensive vacuolization in the tegument and muscle layer, along with shrinkage of glycogen storage cells and depletion of glycogen content (Fig. 7C, D). These structural damages may be associated with the impaired scolex evagination capability, reduced cell viability, and disrupted energy metabolism in PSCs following *P158* KD.

### Transcriptomic signatures of cellular injury and metabolic dysregulation following *P158* KD

**Fig. 8:**
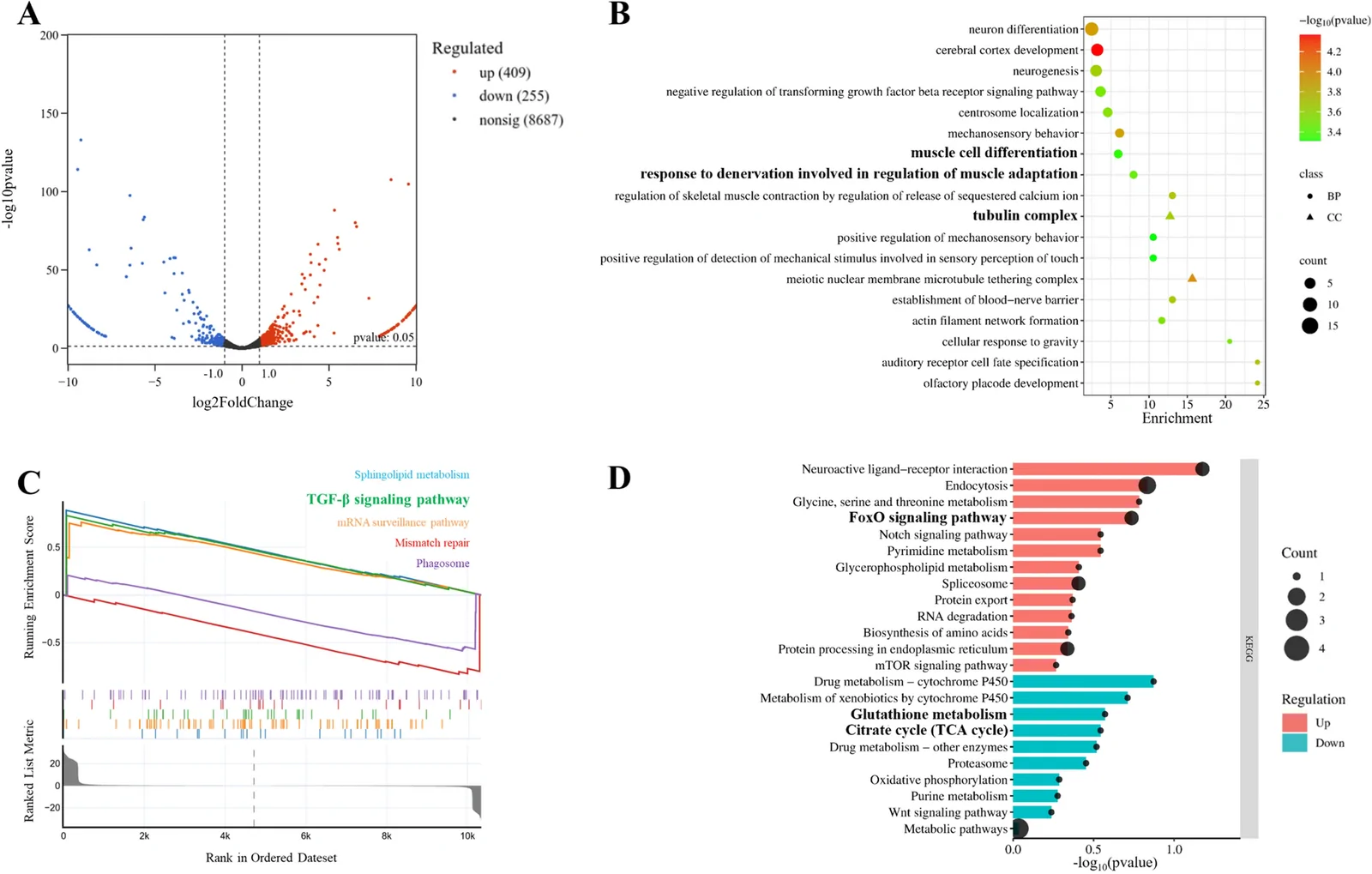
Transcriptomic signatures of cellular injury and metabolic disruption following *P158* KD. **A** Volcano plot showing differentially expressed genes (DEGs) in *P158* KD versus NC groups (|log₂FoldChange|> 1, *P* < 0.05), with 409 upregulated genes (red) and 255 downregulated genes (blue). **B** Gene Ontology (GO) enrichment analysis of DEGs, showing significant enrichment in terms related to muscle cell differentiation, cytoskeletal organization, and contraction regulation. **C** Gene Set Enrichment Analysis (GSEA) plot demonstrating downregulation of the TGF-β signaling pathway. **D** Kyoto Encyclopedia of Genes and Genomes (KEGG) pathway enrichment analysis, showing significant alterations in pathways including the citric acid cycle, glutathione metabolism, and upregulated FoxO signaling

To investigate the molecular mechanisms underlying the effects of *P158* KD on PSC activation and development, we performed RNA sequencing on PSCs of *P158* KD and NC groups. Volcano plot analysis identified 664 significantly differentially expressed genes (|log₂FoldChange|> 1, *P* < 0.05), with 409 upregulated and 255 downregulated (Fig. 8A). GO enrichment analysis showed significant enrichment in terms related to muscle cell differentiation, cytoskeletal attachment, and contraction regulation (Fig. 8B). This suggests abnormal rearrangement of muscle fibers and the cytoskeleton, consistent with the surface microtriches damage and extensive vacuolization in muscle layers observed by SEM and TEM. GSEA revealed overall downregulation of the TGF-β signaling pathway (Fig. 8C), which is critical for germinal cell differentiation and PSC-to-vesicle transition [33]. KEGG enrichment analysis further revealed pathway-level changes potentially associated with the observed phenotypes, including tegumental damage, cytoskeletal/muscle abnormalities, ATP depletion, and impaired evagination/development (Fig. 8D). Enrichment of pathways such as the citric acid cycle and glutathione metabolism was consistent with altered energy metabolism and stress responses, whereas the upregulation of the FoxO signaling pathway may reflect energy stress, nutrient deprivation, or metabolic pressure. These transcriptomic changes are correlative and require further functional validation. Taken together, *P158* KD elicits carried transcriptomic signatures of cellular injury and metabolic dysregulation, likely triggering abnormal scolex evagination, and ultimately impaired activation, defective cyst formation, and cellular structural injury.

## Discussion

Studies have shown that, in in vitro culture systems, scolex evagination in PSCs is an essential step for development toward mature tapeworms. Following evagination induced by conditions such as bile, bile salts, and trypsin, PSCs can further develop into multi-segmented adult worms. In contrast, non-evaginated PSCs or those cultured without bile tend to form vesicular structures, directing development along the intermediate host pathway rather than the adult worm pathway in the definitive host. These experimental conditions mimic the host gastrointestinal environment, confirming that the activation process significantly enhances the developmental capacity and infectivity of PSCs toward the adult stage [34–36]. In animal models, although direct evidence is limited, early studies have indicated that ingested PSCs require activation in the gastrointestinal environment, including scolex evagination induced by pepsin and bile salts, to effectively attach to the intestinal wall and proceed with development. Non-activated PSCs, due to failure in timely scolex evagination and attachment, exhibit reduced infection efficiency in the definitive host [37]. Existing studies have validated that in vitro pre-activated PSCs exhibit enhanced infectivity in animal models or simulated infection systems. For example, in mouse models, PSCs cultured with in vitro activation show stronger infectivity compared to freshly isolated ones [38]. In summary, activation is a critical step for PSC attachment and development into adult worms in the definitive host intestine, playing an important role in the life cycle of *Echinococcus* species and inter-host transmission. Although bile- or bile salt-containing artificial intestinal solution may more closely reproduce the intestinal activation environment of the definitive host, the pepsin-based protocol used here provided a reproducible activation phenotype for functional comparison. Future studies should further validate the role of *P158* under bile-containing intestinal activation conditions.

Based on transcriptomic differences between activated and non-activated PSCs, combined with bioinformatics screening, we identified *P158* as a gene highly expressed following activation. Through loss-of-function studies using RNA interference, we demonstrated for the first time that *P158* is a key functional gene associated with the activation process of *E. multilocularis* PSCs. This gene exhibits specific upregulation upon activation and functions as an independent transcriptional unit distinct from *DDX1*. Functional analyses revealed that *P158* is essential not only for successful PSC activation (scolex evagination) but also for maintaining structural integrity, energy metabolic homeostasis, and normal progression to subsequent developmental stages, exerting multifaceted regulatory roles. These findings establish *P158* as a core molecular switch in the early establishment of infection by *E. multilocularis*, providing new molecular insights into the mechanisms governing protoscolex activation and parasite development in the definitive host.

It is noteworthy that in parasite transcriptome datasets, "expressed protein" is a common annotation tag, typically indicating that the gene has been confirmed to be expressed and likely encodes a protein based on transcriptomic evidence (e.g., RNA-seq reads), although its specific biological function has not yet been experimentally validated or inferred through homology analysis [33, 39]. This situation is prevalent in parasite genomes, as the annotations for many parasites (e.g., *Plasmodium* spp. and *Trypanosoma* spp.) remain incomplete, resulting in a large number of genes being labeled as "expressed protein," "hypothetical protein," or "uncharacterized protein" [40]. In this study, homology alignment revealed high similarity between *P158* and the 5' end sequence of *EgDDX1*. Genomic positioning confirmed that *P158* is located immediately upstream of *EmDDX1* on the same scaffold; however, no long transcript isoform analogous to that of *EgDDX1* was annotated for *EmDDX1*. To clarify their relationship, we experimentally validated the independence of *P158*. ORF prediction indicated virtually no possibility of a continuous coding sequence (CDS) in the intervening spacer region, with the longest ORF comprising only 47 amino acids (144 nucleotides) and all others below 50 amino acids. These short ORFs likely represent random noise, pseudogene remnants, or non-functional sequences lacking translation potential [41]. Additionally, RT-PCR and touchdown PCR results confirmed that *P158* and *EmDDX1* exist as independent transcripts, with the absence of amplification products not attributable to primer issues or low abundance of a potential fusion transcript. RNAi experiments further corroborated their independence at the level of expression regulation and validated the accuracy of the short *EmDDX1* isoform annotation (744 amino acids) in the *E. multilocularis* genome. The *P158* gene shows a significant increase in expression during the activation of PSCs, and its KD leads to pronounced phenotypic changes, indicating that it plays a key role in the activation process of *E. multilocularis* PSCs. Specifically, efficient gene suppression achieved within 1 week is closely associated with markedly reduced scolex evagination rates, lower cyst formation rates, and cellular damage. These findings are further supported by transmission electron microscopy observations of ultrastructural damage: vacuolization in the tegument, increased vacuoles in muscle fibers, glycogen depletion in glycogen storage cells, and cellular atrophy. Notably, the disruption of muscular and tegumental integrity observed upon *P158* KD parallels recent functional studies on fundamental developmental regulators in *E. multilocularis*. For instance, RNAi-mediated silencing of *bcat-1*, the central effector of canonical WNT signaling, similarly results in marked muscle-fiber dysregulation and impaired metacestode vesicle formation [42]. Furthermore, such classic degenerative changes have been widely reported in previous studies, for instance, similar alterations are observed when PSCs of *E. granulosus* PSCs are treated in vitro with nitazoxanide or albendazole (and its metabolites). Such changes are typically regarded as hallmark indicators of drug efficacy, reflecting progressive loss of parasite viability, irreversible degeneration, and eventual death [43]. Taken together, these results and transcriptomic GO enrichment analysis suggest that *P158* might be involved in the tegument remodeling or related signaling pathways required for evagination. Given that scolex evagination entails massive morphogenetic movements and tissue remodeling, the differential expression of genes related to muscle contraction and cytoskeletal organization suggests that *P158* may also govern the intricate cytoskeletal dynamics and mechanical forces necessary to physically execute these profound structural changes [43]. Although our current data do not establish a direct causal relationship between metabolic disruption and failed evagination, the marked ATP depletion and glycogen loss after *P158* KD suggest a plausible functional link. Such an energy deficit may compromise both muscle contraction required for scolex evagination and tegument repair/remodeling during activation.

Analysis using online tools such as DeepLoc2 and TMHMM predicted that *P158* may be a transmembrane protein (Fig. S3), which could be associated with its mechanism of inducing cellular damage. Given the predicted transmembrane feature of P158, we speculate that *P158* may act at the parasite–environment interface during PSC activation. It may function as a membrane-associated sensor or transporter responding to gastrointestinal cues, such as nutrient transporter, or as a signaling scaffold that connects external activation signals with tegument remodeling, cytoskeletal reorganization, and energy metabolism. This model is consistent with the impaired evagination, tegumental damage, ATP reduction, and glycogen depletion observed after *P158* KD. Further studies on P158 localization, interacting partners, and ligand/transport activity will be required to test this hypothesis. Transmembrane proteins have long been important research targets for parasite control. Given the essential role of *P158* in PSC survival and activation, we will further elucidate its protein expression dynamics across developmental stages. Crucially, as PSC activation is an obligate step for the establishment of adult tapeworms in definitive hosts, targeting P158 presents a promising strategy for veterinary interventions (e.g., canine vaccines or specific anthelmintics). Such approaches aim to prevent adult worm maturation and subsequent egg shedding, thereby interrupting the transmission cycle of *E. multilocularis* at its source, offering a proactive strategy for the prevention and control of AE.

## Conclusions

In this study, we identified *P158* as an activation-associated gene that is significantly upregulated during *E. multilocularis* PSC activation. Functional knockdown of *P158* impaired scolex evagination and metacestode vesicle formation, compromised tegumental integrity, and was associated with ATP depletion and glycogen loss. These findings indicate that *P158* plays an important role in PSC activation and early developmental progression, likely through maintaining structural integrity and energy metabolic homeostasis. *P158* may therefore represent a potential target for strategies aimed at interfering with parasite development and transmission.

## Supplementary Information

Supplementary material 1: Figure S1. Amino acid sequence alignment of *P158* with *E. granulosus* *DDX1*. Pairwise alignment of the P158 protein (*E. multilocularis* expressed protein) and *E. granulosus DDX1*. Identical residues are highlighted in red. Alignment generated using NCBI BLASTp (version 2.17.0+; accessed December 2025).

Supplementary material 2: Figure S2. Open reading frame (ORF) prediction in the intergenic spacer between *P158* and *EmDDX1*. Predicted ORFs (>75 nt) identified using NCBI ORF finder (standard genetic code, ATG as start codon only). Translated sequences were subjected to BLASTp searches against the NCBI non-redundant (nr) database (default parameters); no significant homology was detected (E-value > 10^-2^). Schematic generated from ORF finder output, with coordinates relative to the intergenic sequence.

Supplementary material 3: Figure S3. Subcellular localization prediction for P158. Pie chart of predicted localization probabilities generated by DeepLoc 2.0 (accessed December 2025). The highest probability is assigned to the cell membrane (0.5579), followed by lysosome/vacuole (0.4610) and cytoplasm (0.2778). Lower probabilities are shown for other compartments as indicated in the legend.

Supplementary material 4.

## Data Availability

All data generated or analyzed during this study are included in this published article [and its supplementary information files].

## References

[1] Eckert J, Deplazes P. Biological, epidemiological, and clinical aspects of echinococcosis, a zoonosis of increasing concern. Clin Microbiol Rev. 2004;17:107–35. 14726458 10.1128/CMR.17.1.107-135.2004 PMC321468

[2] Liu H, Xie Y, An X, Xu D, Cai S, Chu C, et al. Advances in novel diagnostic techniques for alveolar echinococcosis. Diagnostics (Basel). 2025;15:585. 40075832 10.3390/diagnostics15050585 PMC11898896

[3] Xu X, Qian X, Gao C, Pang Y, Zhou H, Zhu L, et al. Advances in the pharmacological treatment of hepatic alveolar echinococcosis: from laboratory to clinic. Front Microbiol. 2022;13:953846. 36003932 10.3389/fmicb.2022.953846 PMC9393627

[4] Autier B, Robert-Gangneux F, Dion S. Chemotherapy for the treatment of alveolar echinococcosis: where are we? Parasite. 2024;31:56. 39311470 10.1051/parasite/2024055 PMC11418394

[5] Gelmedin V, Caballero-Gamiz R, Brehm K. Characterization and inhibition of a p38-like mitogen-activated protein kinase (MAPK) from *Echinococcus multilocularis*: antiparasitic activities of p38 MAPK inhibitors. Biochem Pharmacol. 2008;76:1068–81. 18789902 10.1016/j.bcp.2008.08.020

[6] Brehm K. The role of evolutionarily conserved signalling systems in *Echinococcus multilocularis* development and host-parasite interaction. Med Microbiol Immunol. 2010;199:247–59. 20376483 10.1007/s00430-010-0154-1

[7] Du X, McManus DP, Fogarty CE, Jones MK, You H. *Schistosoma mansoni* fibroblast growth factor receptor A orchestrates multiple functions in schistosome biology and in the host-parasite interplay. Front Immunol. 2022;13:868077. 35812433 10.3389/fimmu.2022.868077 PMC9257043

[8] Mohammadzadeh T, Sadjjadi SM, Rahimi HR, Shams S. Establishment of a modified *in vitro* cultivation of protoscoleces to adult *Echinococcus granulosus*; an important way for new investigations on hydatidosis. Iran J Parasitol. 2012;7:59–66. 23133473 PMC3488822

[9] Mohammadi MA, Harandi MF, McManus DP, Mansouri M. Genome-wide transcriptome analysis of the early developmental stages of *Echinococcus granulosus* protoscoleces reveals extensive alternative splicing events in the spliceosome pathway. Parasit Vectors. 2021;14:574. 34772444 10.1186/s13071-021-05067-9 PMC8587495

[10] Brehm K, Koziol U. *Echinococcus*-host interactions at cellular and molecular levels. Adv Parasitol. 2017;95:147–212. 28131363 10.1016/bs.apar.2016.09.001

[11] Zheng H, Zhang W, Zhang L, Zhang Z, Li J, Lu G, et al. The genome of the hydatid tapeworm *Echinococcus granulosus*. Nat Genet. 2013;45:1168–75. 24013640 10.1038/ng.2757

[12] Gao H, Bianba Z, Mo X, Hu W, Feng Z, Zhou F, et al. Receptor tyrosine kinase signaling involves *Echinococcus*-host intercommunication: a potential therapeutic target in hepatic echinococcosis. Trop Med Infect Dis. 2024;9:175. 39195613 10.3390/tropicalmed9080175 PMC11360685

[13] Martín-Durán JM, Ryan JF, Vellutini BC, Pang K, Hejnol A. Increased taxon sampling reveals thousands of hidden orthologs in flatworms. Genome Res. 2017;27:1263–72. 28400424 10.1101/gr.216226.116 PMC5495077

[14] Spiliotis M, Brehm K. Axenic *in vitro* cultivation of *Echinococcus multilocularis* metacestode vesicles and the generation of primary cell cultures. Methods Mol Biol. 2009;470:245–62. 19089387 10.1007/978-1-59745-204-5_17

[15] Meng R, Fu Y, Zhang Y, Mou Y, Liu G, Fan H. Indoleamine 2,3-dioxygenase 1 signaling orchestrates immune tolerance in *Echinococcus multilocularis*-infected mice. Front Immunol. 2022;13:1032280. 36439161 10.3389/fimmu.2022.1032280 PMC9691980

[16] Dezaki ES, Yaghoubi MM, Spiliotis M, Boubaker G, Taheri E, Almani PG, et al. Comparison of ex vivo harvested and *in vitro* cultured materials from *Echinococcus granulosus* by measuring expression levels of five genes putatively involved in the development and maturation of adult worms. Parasitol Res. 2016;115:4405–16. 27515372 10.1007/s00436-016-5228-6 PMC5056948

[17] Fabbri J, Elissondo MC. Comparison of different staining methods for determination of viability on *Mesocestoides vogae* tetrathyridia. Parasitol Res. 2019;118:687–92. 30467616 10.1007/s00436-018-6143-9

[18] Herz M, Zarowiecki M, Wessels L, Pätzel K, Herrmann R, Braun C, et al. Genome-wide transcriptome analysis of *Echinococcus multilocularis* larvae and germinative cell cultures reveals genes involved in parasite stem cell function. Front Cell Infect Microbiol. 2024;14:1335946. 38333034 10.3389/fcimb.2024.1335946 PMC10850878

[19] Molgora BM, Rai AK, Sweredoski MJ, Moradian A, Hess S, Johnson PJ. A novel *Trichomonas vaginalis* surface protein modulates parasite attachment via protein:host cell proteoglycan interaction. mBio. 2021;12:e03374-20. 33563826 10.1128/mBio.03374-20 PMC7885099

[20] Mousavi SM, Afgar A, Mohammadi MA, Mortezaei S, Faridi A, Sadeghi B, et al. Biological and morphological consequences of dsRNA-induced suppression of tetraspanin mRNA in developmental stages of *Echinococcus granulosus*. Parasit Vectors. 2020;13:190. 32276648 10.1186/s13071-020-04052-y PMC7146954

[21] Derakhshani A, Mousavi SM, Rezaei M, Afgar A, Keyhani AR, Mohammadi MA, et al. Natural history of *Echinococcus granulosus* microcyst development in long term *in vitro* culture and molecular and morphological changes induced by insulin and BMP-4. Front Vet Sci. 2023;9:1068602. 36699324 10.3389/fvets.2022.1068602 PMC9868913

[22] Liu Z, Guo X, Guo A, Zhang S, Zou Y, Wang Y, et al. HIV protease inhibitor nelfinavir is a potent drug candidate against echinococcosis by targeting Ddi1-like protein. EBioMedicine. 2022;82:104177. 35843171 10.1016/j.ebiom.2022.104177 PMC9294487

[23] Livak KJ, Schmittgen TD. Analysis of relative gene expression data using real-time quantitative PCR and the 2−ΔΔCT method. Methods. 2001;25:402–8. 11846609 10.1006/meth.2001.1262

[24] Albani CM, Fuentes G, Ramírez CL, Pensel PE, Gatti F, Albanese A, et al. Anthelmintic effect of cannabidiol against *Echinococcus granulosus* sensu stricto. Trop Med Infect Dis. 2024;9:35. 38393124 10.3390/tropicalmed9020035 PMC10892078

[25] Love MI, Huber W, Anders S. Moderated estimation of fold change and dispersion for RNA-seq data with DESeq2. Genome Biol. 2014;15:550. 25516281 10.1186/s13059-014-0550-8 PMC4302049

[26] Yu G, Wang LG, Han Y, He QY. ClusterProfiler: an R package for comparing biological themes among gene clusters. OMICS. 2012;16:284–7. 22455463 10.1089/omi.2011.0118 PMC3339379

[27] Thompson RC. Growth, segmentation and maturation of the British horse and sheep strains of *Echinococcus granulosus* in dogs. Int J Parasitol. 1977;7:281–5. 924713 10.1016/0020-7519(77)90036-4

[28] Ritler D, Rufener R, Sager H, Bouvier J, Hemphill A, Lundström-Stadelmann B. Development of a movement-based *in vitro* screening assay for the identification of new anti-cestodal compounds. PLoS Negl Trop Dis. 2017;11:e0005618. 28520724 10.1371/journal.pntd.0005618 PMC5448807

[29] Tsai IJ, Zarowiecki M, Holroyd N, Garciarrubio A, Sanchez-Flores A, Brooks KL, et al. The genomes of four tapeworm species reveal adaptations to parasitism. Nature. 2013;496:57–63. 23485966 10.1038/nature12031 PMC3964345

[30] Olson PD, Tracey A, Baillie A, James K, Doyle SR, Buddenborg SK, et al. Complete representation of a tapeworm genome reveals chromosomes capped by centromeres, necessitating a dual role in segregation and protection. BMC Biol. 2020;18:165. 33167983 10.1186/s12915-020-00899-w PMC7653826

[31] Galindo M, Schadebrodt G, Galanti N. *Echinococcus granulosus*: cellular territories and morphological regions in mature protoscoleces. Exp Parasitol. 2008;119:524–33. 18508050 10.1016/j.exppara.2008.04.013

[32] Chaudhry S, Zurbriggen R, Preza M, Kämpfer T, Kaethner M, Memedovski R, et al. Dual inhibition of the *Echinococcus multilocularis* energy metabolism. Front Vet Sci. 2022;9:981664. 35990276 10.3389/fvets.2022.981664 PMC9388906

[33] Kaethner M, Epping K, Bernthaler P, Rudolf K, Thomann I, Leitschuh N, et al. Transforming growth factor-β signalling regulates protoscolex formation in the *Echinococcus multilocularis* metacestode. Front Cell Infect Microbiol. 2023;13:1153117. 37033489 10.3389/fcimb.2023.1153117 PMC10073696

[34] Maimaitizunong P, Li J, Wu C, Tian M, Qi W, Jiao H, et al. Protoscolex evagination and pre-worm maintenance with bile are key processes for adult worm development of *Echinococcus granulosus* and *Echinococcus multilocularis in vitro*. Res Sq. 2022. 10.21203/rs.3.rs-2208857/v1.

[35] Smyth JD, Davies Z. *In vitro* culture of the strobilar stage of *Echinococcus granulosus* (sheep strain): a review of basic problems and results. Int J Parasitol. 1974;4:631–44. 4609935 10.1016/0020-7519(74)90028-9

[36] Debarba JA, Monteiro KM, Moura H, Barr JR, Ferreira HB, Zaha A. Identification of newly synthesized proteins by *Echinococcus granulosus* protoscoleces upon induction of strobilation. PLoS Negl Trop Dis. 2015;9:e0004085. 26393918 10.1371/journal.pntd.0004085 PMC4578768

[37] Smyth JD. *In vitro* studies and host-specificity in *Echinococcus*. Bull World Health Organ. 1968;39:5–12. 5303841 PMC2554377

[38] Zhang WB, Jones MK, Li J, McManus DP. *Echinococcus granulosus*: pre-culture of protoscoleces *in vitro* significantly increases development and viability of secondary hydatid cysts in mice. Exp Parasitol. 2005;110:88–90. 15804383 10.1016/j.exppara.2005.02.003

[39] Olson PD, Zarowiecki M, James K, Baillie A, Bartl G, Burchell P, et al. Genome-wide transcriptome profiling and spatial expression analyses identify signals and switches of development in tapeworms. EvoDevo. 2018;9:21. 30455861 10.1186/s13227-018-0110-5 PMC6225667

[40] Parsons M, Myler PJ. Illuminating parasite protein production by ribosome profiling. Trends Parasitol. 2016;32:446–57. 27061497 10.1016/j.pt.2016.03.005 PMC4884476

[41] Couso JP, Patraquim P. Classification and function of small open reading frames. Nat Rev Mol Cell Biol. 2017;18:575–89. 28698598 10.1038/nrm.2017.58

[42] Herrmann R, Herz M, Rudolf K, Koike A, Spiliotis M, Bergmann M, et al. Canonical WNT signalling governs *Echinococcus* metacestode development. PLoS Pathog. 2026;22:e1014046. 41871170 10.1371/journal.ppat.1014046 PMC13029709

[43] Shi Q, Liu C, Huo L, Tao Y, Zhang H. Silencing TUBB3 expression destroys the tegument and flame cells of *Echinococcus multilocularis* protoscoleces. Animals (Basel). 2022;12:2471. 36139331 10.3390/ani12182471 PMC9495074

